# Recent Wetting and Glacier Expansion in the Northwest Himalaya and Karakoram

**DOI:** 10.1038/s41598-017-06388-5

**Published:** 2017-07-21

**Authors:** Ram R. Yadav, Anil K. Gupta, Bahadur S. Kotlia, Vikram Singh, Krishna G. Misra, Akhilesh K. Yadava, Anoop K. Singh

**Affiliations:** 1 Wadia Institute of Himalayan Geology, Dehradun, India; 2 Department of Geology & Geophysics, Indian Institute of Technology, Kharagpur, India; 3 Centre of Advanced Study in Geology, Kumaun University, Nainital, India; 4 Birbal Sahni Institute of Palaeosciences, 53 University Road, Lucknow, India

## Abstract

Hydroclimatic variability driven by global warming in the climatically vulnerable cold semi-arid to arid northwest (NW) Himalaya is poorly constrained due to paucity of continuous weather records and annually resolved proxies. Applying a network of annually resolved tree-ring-width chronologies from semi-arid region of Kishtwar, Jammu and Kashmir, India, we reconstructed April-May standardized precipitation index extending back to A.D. 1439 (576 years). The reconstructed series is featured by the most conspicuous long-term droughts during the 15^th^ to early 17^th^ centuries followed by a general wetting, with 1984–2014 being the wettest interval in the past 576 years. The data, consistent with other independently developed tree-ring-based hydrological records from cold semi-arid to arid NW Himalaya and Karakoram, point to an increased regional wetting in the recent decades. Such an increased wetting might have led to the anomalous behaviour of glaciers in the NW Himalaya and Karakoram in contrast to the general receding trends in the central and eastern Himalaya.

## Introduction

Glaciers in the NW Himalaya and Karakoram, a perennial source of stream recharge, largely sustain on winter and spring (December–May) precipitation brought by mid-latitude westerlies^1, 2^. Most of the glaciers in different parts of the Earth show receding trends owing to global warming, however, some glaciers in the NW Himalaya and Karakoram show anomalous trend with stability or mass gain in the recent decades^3–8^, accentuating the importance of changing regional precipitation patterns^9^ in response to global climate change. Nevertheless, our understanding on spatio-temporal variability of precipitation, as one of the most important drivers for the cryospheric change, remains poorly constrained due to non-availability of long and continuous precipitation records^10^. We investigated April-May standardized precipitation index variability in semi-arid Kishtwar in the NW Himalaya reconstructed from a network of annually resolved Neoza pine (*Pinus gerardiana* Wall ex Lamb.) and Himalayan cedar (*Cedrus deodara* (Roxb.) G. Don) ring-width chronologies from a network of moisture stressed sites. The precipitation records of Kishtwar (1901–50) show that ~66% of the annual precipitation occurs from December to May of which ~27% falls in April-May alone, connoting its significant contribution to the regional hydrology.

Observational precipitation records from the NW Himalaya and Karakoram, though very short and scanty, are restricted to valley floors, and show mixed, both increasing/decreasing trends that indicate orography-forced high spatial variability^11–17^. Tree-ring-based precipitation records from cold semi-arid to arid NW Himalaya and Karakoram have revealed increased pluvial conditions in the 20^th^ century^18–20^. However, hitherto Jammu and Kashmir in the NW Himalaya represents a data void, and thus annually resolved tree-ring records are essentially required to understand hydroclimatic variability across the north-south transect from Karakoram to the NW Himalaya where major part of annual precipitation occurs in winter and spring under the influence of mid-latitude westerlies. Tree-ring-based hydroclimatic proxy records^21–25^ from Jammu and Kashmir are short and do not show significant variations on inter-decadal time scale. Such studies in this region after the initial attempts in the 1980s^26–29^ could not take pace further largely due to lack of logistics and difficulties experienced in approaching remote locations, where pristine old forest stands are found. Here, we present analyses of standardized precipitation index of April-May (SPI2-May) reconstructed for the first time using network of annually resolved tree-ring data of Neoza pine and Himalayan cedar from semi-arid Padder Valley of Kishtwar, Jammu and Kashmir, NW Himalaya. The data were compared with other proxy records available from cold semi-arid to arid regions of the NW Himalaya and Karakoram to understand spatio-temporal variability in droughts vis-à-vis the state of glaciers in long-term perspective. High-resolution archives of hydroclimate from a close network of orographically varied Himalayan regions, as presented here, should also be useful in assessment of sensitivity of precipitation to a range of forcing factors on different timescales and also evaluate climate model skills in projecting future trend of climate variability^30^.

## Results

### Moisture sensitive tree-ring data

**Figure 1:**
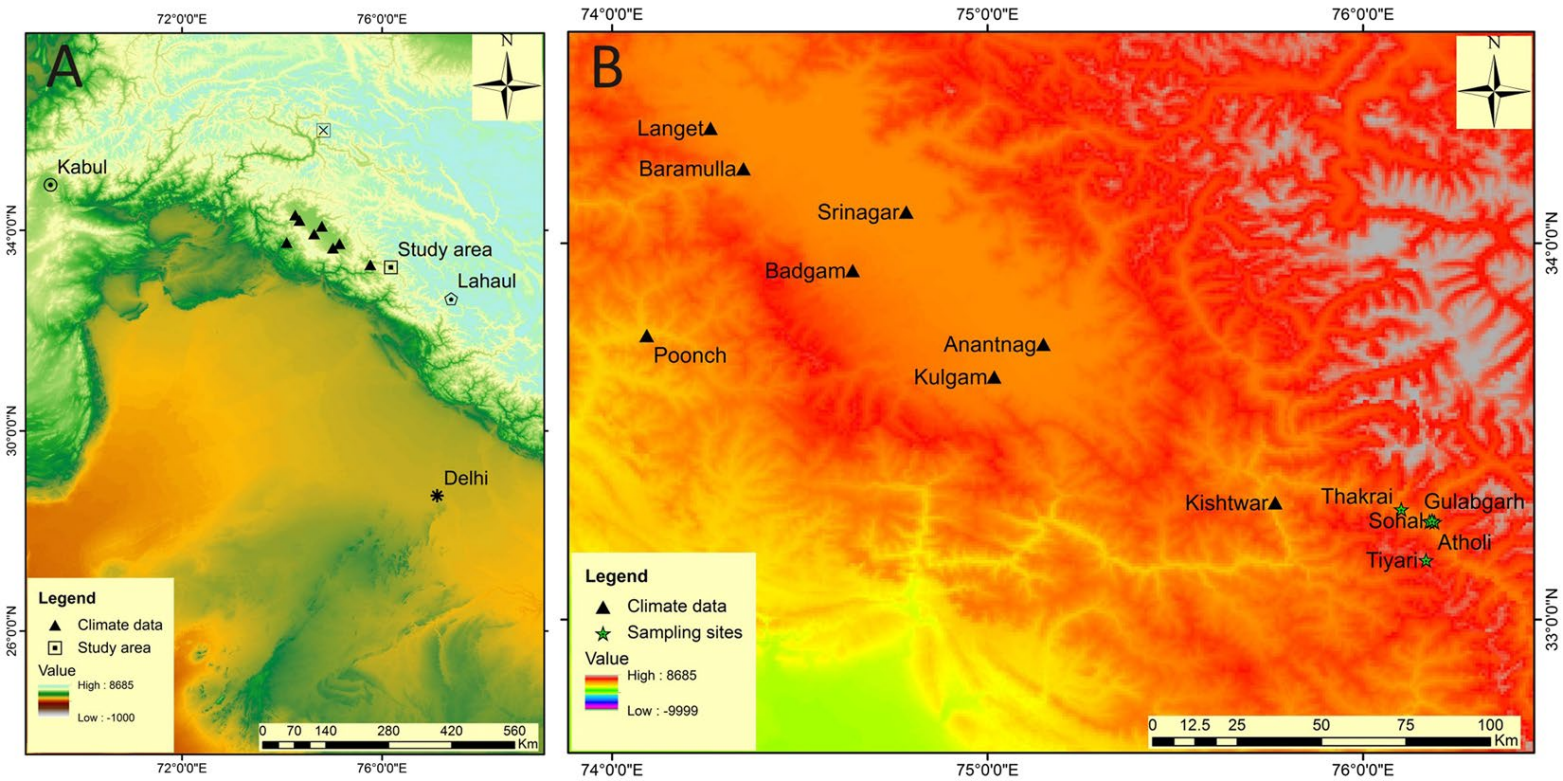
Site locations, A- tree-ring sites (square with dot in centre), and proxy locations used in comparison (Lahaul, polygon with dot^19^; Karakoram, crossed square^18^; B-Detailed location of tree-ring sampling sites and meteorological stations used in this study. The figure was generated using the software ArcGIS 10.3.

Pristine stands of Neoza pine and Himalayan cedar growing at moisture stressed sites (four and one site of each species respectively) distributed over an altitude range of 1900–2400 m asl in Kishtwar (Fig. 1, Supplementary Table 1) were sampled in early August, 2015. We underlined on collection of increment cores of these species as their ages in cold-arid regions of the NW Himalaya are known to extend over the millennium^20, 31, 32^. The Neoza pine trees usually prefer to colonize very dry rocky settings and for such ecological preferences it often grows pure and scattered forming open canopy forest. The competition among trees in such open stands is low making them a sensitive sensor of hydroclimatic variability. The growth pattern analyses of trees from open stands indicate radial growth increase in recent decades, especially since 1970s (Supplementary Fig. 1). The growth ring sequences in increment core samples of Neoza pine and Himalayan cedar were precisely crossdated and ring-width chronologies prepared using established signal-free standardization method^33^. Two types of ring-width chronologies, one by applying cubic spline detrending with a 50% response wavelength of 2/3^rd^ of the length of each ring-width series^34^, and another by Regional Curve Standardization of ring-width series of Neoza pine samples possessing pith^35, 36^ were developed (Supplementary Table 1; Supplementary Figs 2–4). Expressed population signal statistics^37^ was used to identify population signal and length of chronologies for climate studies. The ring-width chronologies revealed significant correlations (Supplementary Table 2) as well as consistency in year-to-year, inter-decadal and centennial scale variability (Supplementary Fig. 4), underpinning a strong common climate signal.

### Climate data

Weather records from the NW Himalaya as a whole and Jammu and Kashmir in particular are very patchy and sparse. The operational weather stations are mainly localized in valley floors, which do not represent climate of the tree ring sites, located usually at remote, high elevation sites in the Himalaya. Padder Valley of Kishtwar district is situated in the northeast corner of Jammu region in the Pir Panjal Range of Jammu and Kashmir, where influence of summer monsoon (June–September) is low, contributing ~16% of the annual precipitation (Supplementary Fig. 5). A major portion of precipitation occurring in winter and spring (December–May) is brought by mid-latitude westerlies. Kishtwar receives the lowest precipitation across the entire Jammu and Kashmir region, with annual rainfall of ~822 mm. Due to low average annual precipitation the whole of Kishtwar has been declared drought prone^38^. Weather recording of numerous stations in Jammu and Kashmir was discontinued after the partition of India in 1947. The only weather station with a long, continuous record is Srinagar with data extending back to 1893. The geodesic distance between Srinagar and Kishtwar is ~122 km, and both locations have similar precipitation regime. However, high precipitation variability in the orography dominated Himalayan region within short distance makes it difficult and also unreasonable to calibrate chronologies with single station data originating far from the tree-ring sampling sites. The gridded precipitation data from the high-elevation NW Himalaya, in many cases distinctly differing from the *in-situ* weather records (Supplementary Fig. 6) are not suitable for calibration of tree-ring chronologies. In view of this we prepared a mean regional precipitation series by merging eight station homogeneous datasets (Supplementary Fig. 7, Supplementary Table 3) to calibrate tree- ring chronologies. However, in case of temperature which shows large scale consistency, single station data of Srinagar extending back to 1893 were used.

### Climate signal in ring-width chronologies

**Figure 2:**
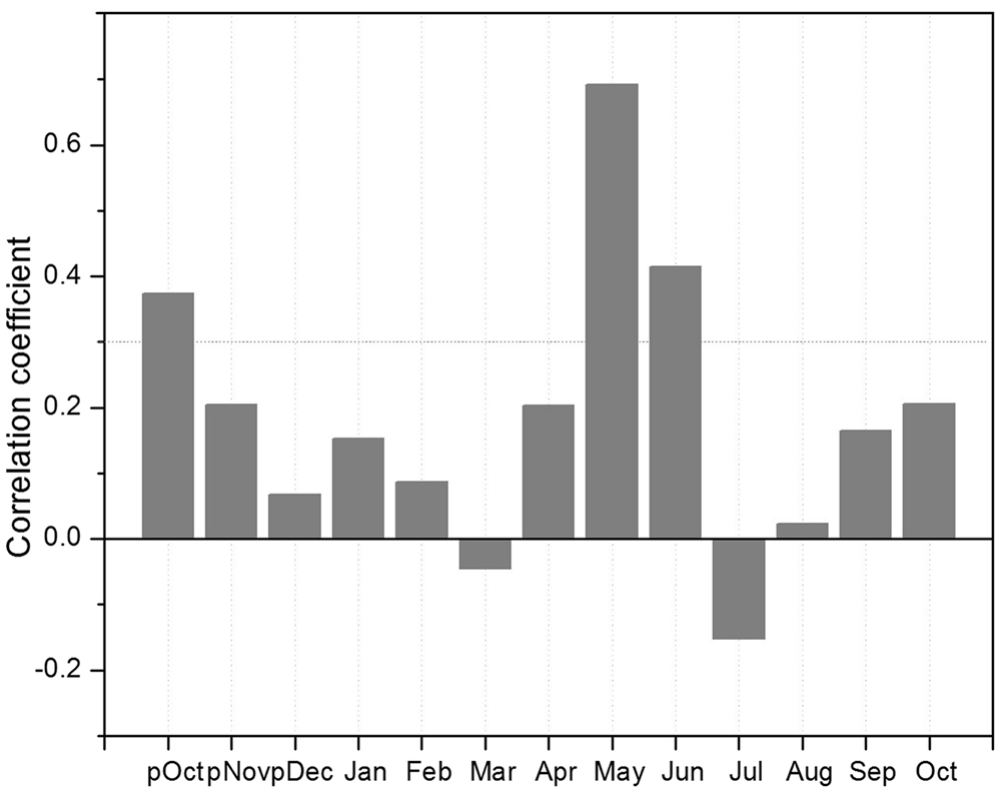
Correlation between PC#1 of ring-width chronologies calculated for the common chronology period and monthly SPI2 values for the period 1907–1946. The dotted horizontal line is 95% confidence limit.

Pearson correlation coefficients and principal component analyses (PCA) of ring-width chronologies (four Neoza pine and one Himalayan cedar) for the common period A.D. 1717–2014 indicated high common variability and strength of common forcing factor (Supplementary Table 2). The bootstrapped correlation analyses of ring-width chronologies and PC#1 (eigenvalue 3.8) with climate variables, viz., regional precipitation series and temperature of Srinagar were performed for different periods using the program DENDROCLIM2002^39^ (Supplementary Fig. 8A–F). Monthly climate data for a window from September of the previous growth year to October of current year were used in correlation analyses. The highest correlations were observed with precipitation for the period 1907–1946, when mean precipitation series contained seven station data sets (Supplementary Table 3). Correlation analyses indicated that precipitation of current year April and May showed strongest, direct relationship with Neoza pine as well as Himalayan cedar chronologies and PC#1 (Supplementary Fig. 8A–F). The mean monthly temperature of summer months showed negative relationship with the chronologies and PC#1, which were usually stronger in May and June (Supplementary Fig. 8A–F). We observed that correlation of ring-width chronologies as well as PC#1 of Neoza pine and Himalayan cedar with monthly precipitation and temperature variables nearly reflects mirror image of each other (Supplementary Fig. 8A–F) indicating that cool and moist conditions in April-May favour tree growth over semi-arid sites in Kishtwar. Considering these findings we studied relationship between PC#1 and commonly used drought indices, viz., self-calibrating Palmer Drought Severity Index (scPDSI)^40^, Standardized Precipitation Evapotranspiration Index (SPEI)^41^ and Standardized Precipitation Index (SPI)^42^. Pearson correlation analyses of SPI with tree-ring chronologies (figure not shown) as well as PC#1 showed highest correlation with the SPI of April and May (SPI2-May) (Fig. 2; Supplementary Fig. 9), which was reconstructed using a linear regression model. We used a nested approach^43^ to optimize the reconstruction length as the number of available chronologies decreased back in time (see methods). Calibration and verification statistics, such as Pearson correlation coefficient, sign test, reduction of error (RE), and coefficient of efficiency (CE)^44^ denoted statistical skill in the reconstruction (Supplementary Table 4). The consistency in SPI2-May reconstructions developed using chronologies prepared by applying two independent detrending methods (spline and RCS) (r = 0.93, 1544–2014; Supplementary Fig. 10) affirmed fidelity of low frequency variations in our data. In view of this, we present analyses of SPI2-May series, which was developed using nested approach (A.D. 1439–2014) where a network of five chronologies was used. To understand the presence of regional-scale signatures in SPI2-May reconstruction we performed field correlations using gridded scPDSI^40^ available through the KNMI climate explorer (https://climexp.knmi.nl^45^), and PDSI of Monsoon Asia Drought Atlas (MADA^46^). The SPI2-May reconstruction showed consistency with the corresponding month’s drought variability over large parts of the Himalaya-Karakoram and Central Asia (Supplementary Fig. 11). The 11-year running mean of SPI2-May and April-May scPDSI of coordinate close to Kishtwar (32.5°–35°N and 75°–77.5°E) also showed good consistency (Supplementary Fig. 12). The SPI2-May reconstruction displayed strong direct association with June-July-August PDSI of MADA^46^ over the High Asia mountain regions (Supplementary Fig. 13) as well endorsing regional scale signatures in our reconstruction.

## Discussion

**Figure 3:**
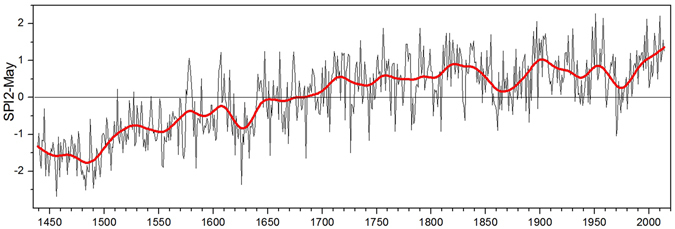
SPI2-May reconstructed series (A.D. 1439–2014). The thick line superimposed on the reconstruction is 40-year low pass filter.

**Figure 4:**
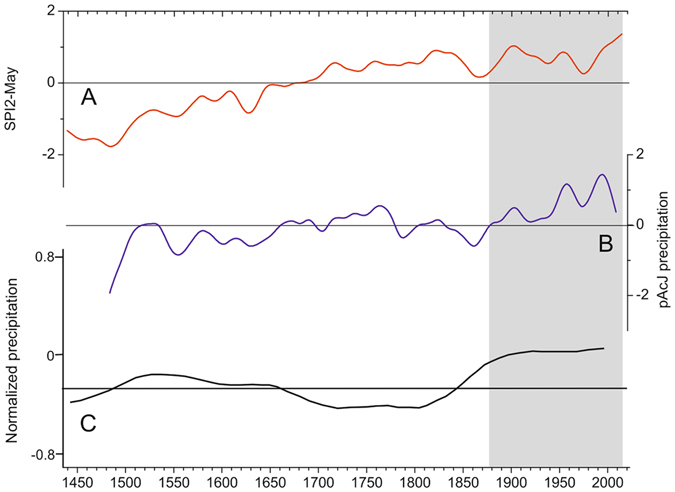
Tree-ring-based hydroclimatic records from the northwest (NW) Himalaya, India and Karakoram, northern Pakistan showing increased wetting in the 20^th^ century and recent decades. A- SPI2-May reconstruction (present study), B-tree-ring-based previous year August to current year July (pAcJ) precipitation for cold-arid Lahaul, NW Himalaya^19^, the data in A and B were normalized relative to the mean and standard deviation of the length of respective series and 40-year spline filtered; C- tree-ring δ^18^O based precipitation for Karakoram, northern Pakistan (Reprinted by permission from Macmillan Publishers Ltd: [Nature]^18^, copyright (2006). The data represent 150 year spline filter. The horizontal line is mean of reconstruction (A.D. 950–1990).

Our SPI2-May reconstruction revealed high inter-annual, inter-decadal-to-centennial scale variability with distinct wetting since early part of the reconstruction (Fig. 3). Long-term variability observed in our data, consistent with different hydroclimate proxies of reliable chronologies from arid Central Asia^47^, where precipitation is largely controlled by the mid-latitude westerlies, support large-scale wet Little Ice Age and increasing pluvial phase since 1980s^47^. The SPI2-May reconstruction exhibited pronounced century scale excursions with usually negative values (drier climate) from A.D. 1439–1660s and positive excursions (wetter climate) since A.D. 1670s onwards. The long-term pluvial phase from 1670s to 2014 was also punctuated by relatively drier periods of 1850s–1870s and 1970s. A comparison of SPI2-May reconstructed series with precipitation records developed from cold-arid regions of Lahaul^19^ and Kinnaur^20^ in the NW Himalaya, where annual precipitation variability is largely controlled by the mid-latitude westerlies in winter and spring, revealed strong consistency in variations over inter-annual, inter-decadal and centennial timescale (Supplementary Fig. 14). The credibility of our SPI2-May reconstruction is further supported by the existence of significant correlations with these precipitation reconstructions (Lahaul- previous year August to current July; r = 0.63, A.D. 1439–2008, two tailed p < 0.0001 and Kinnaur- March-April-May; r = 0.48, A.D. 1439–2011, two tailed p < 0.0001) (Supplementary Fig. 14). The correlations of SPI2-May with the above time series during first and second half of the 20^th^ century were also stable and significant (r = 0.66, 1901–1946, r = 0.75, 1947–2008 with pAcJ precipitation of Lahaul and r = 0.48, 1901–1946, r = 0.49, 1947–2011 with MAM precipitation of Kinnaur). Distinct consistency in tree-ring-based hydroclimatic records from the NW Himalaya and Karakoram (Fig. 4) bespeaks regional wetting during winter and spring in the 20^th^ and early 21^st^ centuries, which could be associated with anomalous behaviour of glaciers unlike their counterparts in summer monsoon fed glaciers of the central and eastern Himalaya.

The most conspicuous feature of the reconstructed drought indices is an increase in SPI values in the late 20^th^ to early 21^st^ century, with 1984–2014 being the wettest 31-year of the past 576 years (A.D. 1439–2014) (Supplementary Table 5). The increased wetting tendency noted in recent decades is consistent with the strengthening of western disturbances in the NW Himalaya and Karakoram^48^. Snow cover measurements of May in Kashmir during 2004–2012 have also shown an increasing trend^14^ pointing towards strengthening of the influence of the westerlies. Consistent with our data, the weather records from arid regions of Central Asia, where precipitation is controlled by the mid-latitude westerlies, have also shown significant increase in precipitation in recent decades^49^. The decreasing trend in dust storms in Central Asia (1936–2005)^50^ could also be linked with the increasing trend in precipitation in the region. The stability/advancement of many large glaciers in Lahaul^51^ and Karakoram^3–8^ could be associated with the increased import of moisture by the mid-latitude westerlies in recent decades. The reduction in run-off of Hunza and Shyok river flow during 1961–2000 has also been implicated to glacier stability, which has been found to be largely due to increasing winter precipitation^13^. These features are also consistent with the descending trend in modelled equilibrium-line altitude reported in Karakoram during 1976–1995^52^. The glacier thickening in the northwest Himalaya and Karakoram is in agreement with the increasing trend in precipitation^11^, but strikingly in contrast to mass balance studies from the central and eastern Himalaya^6, 8, 52–56^ where glaciers are in recession phase.

The 20^th^ century featured by pluvial conditions was also punctuated by drier 1970s with 1970 and 1971 being very dry. The hydrological records available from semi-arid regions of the NW Himalaya show that these droughts were wide spread in cold-arid regions of Lahaul-Spiti^19, 57^ and Kinnaur^20^. The extended large-scale droughts of 1970s could have caused serious stress on socio economy of the Kishtwar region. The droughts of 1970–71 were widespread in Afghanistan, Pakistan and Tajikistan as well, largely due to failure of winter snowfall triggering population displacement, loss of cattle and severe food shortages^58^. To understand the causal factors of such droughts we investigated relationship of SPI data with North Atlantic Oscillation (NAO) and El Nino Southern Oscillation (ENSO), which are prominent modes of variability exerting strong influence on precipitation in southwest central Asia and north India^11, 59, 60^. The positive phase of NAO and warm phase of ENSO are associated with positive anomalies in precipitation, however, the former has strong linkage with precipitation variability in winter only when it is most active^11, 59, 60^. Our SPI2-May data did not reveal significant relationship with NAO except weak correlation with the indices of April only (r = 0.12, p = 0.341, 1950–2014). We observed that the SPI data are directly associated with temperature anomalies in the tropical eastern Pacific Ocean and Indian Ocean (Supplementary Figs 15–18). Consistent with this finding, SST anomaly plots for the dry years 1970 and 1971 indicated −0.4 K cooler SSTs in the eastern tropical Pacific Ocean (Supplementary Fig. 18). Cold SSTs in the eastern Pacific Ocean (La Nina phase of ENSO) were reported earlier to be associated with precipitation failure in southwest central Asia under the dominant influence of western disturbances^61^.

In conclusion we would like to emphasize that our SPI reconstruction from semi-arid region of Kishtwar in combination with other tree-ring-based hydroclimatic records from cold semi-arid to arid regions of the NW Himalaya and Karakoram indicate increased wetting in recent decades (Fig. 4). Such an increased wetting could be augmenting the positive glacier mass balance in the NW Himalaya and Karakoram. However, valley specific aspects and orographic features could be responsible for varying response of glaciers to climate change. The increasingly wetter conditions observed in cold semi-arid to arid regions of the NW Himalaya and Karakoram in the 21^st^ century are of high socioeconomic relevance, as precipitation in these areas, to a large extent, controls the discharge of a dense network of rivers, which are lifeline to the large up and down stream population in the region. The findings presented here are also in agreement with the pluvial 21^st^ century in the NW Himalaya and Karakoram depicted in climate model projections^62, 63^. However, at this stage we also express some reservation on the magnitude of tree-ring depicted wetting in recent decades as the enhancement of tree growth could also be partly driven by increased water use efficiency in trees with increasing level of atmospheric CO_2_ and anthropogenic nitrogen deposition^64, 65^.

Our study is novel as it presents the first comprehensive tree-ring-based precipitation record indicating increase in winter and spring precipitation in the Indian NW Himalaya which has augmented the growth of glaciers in the Jammu and Kashmir and Karakoram region during the past decades. This observation is in contrast to the behaviour of glaciers in the adjacent states of Himachal Pradesh and Uttarakhand in the Indian Himalaya, where most of the glaciers have been waning at a fast rate. This study thus breaks the myth that all the glaciers in the Himalayan region have been receding as one block with increased global warming. Our study is of great socio-economic relevance as Himalayan glacier melt water feeds the perennial rivers of the NW Himalaya that cater to the drinking and irrigation water needs of the region.

## Methods

### Tree-ring chronology development

The tree-ring samples of Neoza pine and Himalayan cedar in the form of increment cores were collected from moisture stressed sites in Padder valley, Kishtwar, Jammu and Kashmir, NW Himalaya. Each growth ring in the samples was assigned to the calendar year of its formation by using established procedures of crossdating. The ring-widths in precisely dated increment core samples were measured to the nearest 0.01 mm using a LINTAB system (Rinntech, Heidelberg, Germany). The ring-width measurement series were standardized using appropriate detrending methods to maximize the common variance and remove non-climatic growth trends. In this process, signal free detrending^33^ by applying cubic spline with a 50% response wavelength of 2/3^rd^ of the length of each ring-width series^34^, and the Regional Curve Standardization of ring-width measurement series of samples possessing pith^35, 36^ were used. The selection of chronology length was based on expressed population signal (EPS) statistics commonly practiced in dendroclimatology^37^.

### Climate data

As weather records from any station close to tree ring sites is not available, we prepared regional precipitation series by merging monthly precipitation data of eight weather stations in Jammu and Kashmir (Supplementary Table 3). A regional monthly data series of precipitation was developed by combining the z-scores calculated with respect to mean and standard deviation of the common period (1916–1946). The mean regional monthly precipitation series were then rescaled to total precipitation in millimetres with respect to the average of mean and standard deviation of respective months of all eight stations. The regional precipitation series was used in the computation of the standardized precipitation index^42^.

### SPI2-May Reconstruction

The network of five ring-width chronologies (four Neoza pine and one Himalayan cedar) was used to calibrate and reconstruct SPI2-May. At first, the predictor chronology variables (t0 and t + 1) were tested for their relationships with SPI2-May. We observed that only t0 variable of the chronologies passed the threshold correlation significance level (p ≤ 0.01). As the number of available tree-ring chronologies decreased back in time we used a nested approach^43^ to maximize the reconstruction length. In this procedure the principal components calculated for each nest with a decreasing number of predictor chronologies (t0) back in time were used in the reconstruction. The PC#1 with an eigenvalue >1 of each nest showing a significant positive relationship with SPI2-May (i.e., 1907–1946) was used in calibration and reconstruction. The split period calibration in 1907–1926 and 1927–1946 approach was performed to test the fidelity of relationship between PC#1 and SPI2-May. Due to short length of observed SPI2-May series of only 40 years (1907–1946), we also performed leave-one-out cross-validation^66^ to test the validity of calibration model. After establishing the fidelity of calibration model we used 1907–1946 regression model to develop the final reconstruction for each nest. Accordingly, four reconstructions of different lengths involving a varying number of predictor chronologies in different nests were developed. The nested reconstructions developed using the full period (1907–1946) calibrations were spliced together to develop the final reconstruction extending from A.D. 1439 to 2014. To minimize possible artifacts associated with the changes in variance through time due to decreasing number of predictors, the mean and standard deviation of each nested series was scaled to that of the most replicated nest one (A.D. 1717–2014) prior to averaging.

## Electronic supplementary material

Supplementary Information
