# Supplementary material for: Recent Wetting and Glacier Expansion in the Northwest Himalaya and Karakoram
Source: Sci Rep. 2017 Jul 21;7:6139. doi: 10.1038/s41598-017-06388-5 (PMC5522409; doi:10.1038/s41598-017-06388-5)
Supplement: Supplementary file 1 — Supplementary Information [file 41598_2017_6388_MOESM1_ESM.pdf]

Supplementary data

RECENT WETTING AND GLACIER EXPANSION IN THE NORTHWEST HIMALAYA AND KARAKORAM

Ram R Yadav[a,*], Anil K Gupta[a,¥], Bahadur S. Kotlia[b], Vikram Singh[c], Krishna G. Misra[c], Akhilesh K. Yadava[c], Anoop K. Singh[b]

[a]Wadia Institute of Himalayan Geology, Dehradun, India and [¥]Department of Geology & Geophysics, Indian Institute of Technology, Kharagpur - 721 302, India
[b]Centre of Advanced Study in Geology, Kumaun University, Nainital, India
[c]Birbal Sahni Institute of Palaeosciences, 53 University Road, Lucknow, India

*Corresponding author Ram R. Yadav: rryadav2000@gmail.com
Anil K. Gupta: anilgupta021160@gmail.com
Bahadur S. Kotlia: bahadur.kotlia@gmail.com
Vikram Singh: vnegi850@gmail.com
Krishna G. Misra: kgmisrabsip@gmail.com
Akhilesh K. Yadava: akhilesh.k.yadava@gmail.com
Anoop K. Singh: anoop1818@gmail.com

Supplementary Table 1. Site location and length of chronologies with number of samples used*. Himalayan cedar (*Cedrus deodara*) trees in Sohal were found growing just above the Neoza pine zone.

S.No.	Sites	Location	Species	Number of cores/trees	Chronology span (A.D.)	Chronology span with EPS>0.85
1	Sohal	33°15'48.2"N76°11'02.3"E	*Pinus gerardiana*	64/36	1257-2014	1383-2014
2	Gulabgarh	33°15'35.4"N 76°11'24.0"E	*Pinus gerardiana*	65/36	1259-2014	1439-2014
3	Thakrai	33°17'36.0"N 76°06'07.2"E	*Pinus gerardiana*	27/18	1353-2014	1483-2014
4	Atholi	33°15'40.1"N76°10'38.5"E	*Pinus gerardiana*	42/24	1500-2014	1534-2014
5	Sohal	33°15'48.2"N76°11'02.3"E	*Cedrus deodara*	16/11	1509-2014	1717-2014
6		RCS Chronology	*Pinus gerardiana*	52/45	1259-2014	1544-2014

*Details of site locations are shown in Figure 1. EPS - expressed population signal

Supplementary Table 2. Pearson correlation analyses among the site and species chronologies developed from Kishtwar, northwest Himalaya for the common period (A.D. 1717-2014). The chronologies are of Neoza pine except one of Himalayan cedar from Sohal as indicated in parenthesis.

Sites/Chronology	Sohal	Gulabgarh	Thakrai	Atholi	Sohal (Himalayan cedar)
Gulabgarh	0.895				
Thakrai	0.829	0.786			
Atholi	0.772	0.847	0.815		
Sohal (Himalayan cedar)	0.689	0.704	0.642	0.672	
RCS	0.938	0.862	0.771	0.724	0.682

Supplementary Table 3. Details of meteorological stations of which available precipitation data were used to develop mean regional precipitation series.

Station	Latitude	Longitude	Altitude (m)	Record length
Anantnag	33º 43′ 53.49″	75º 08′55.95″	1600	1901-1952
Badgam	33º 55′ 39.31″	74º 38′ 26.68″	2116	1901-1947
Baramulla	34º 11′ 55.47″	74º 20′ 59.82″	1613	1902-1957
Kishtwar	33º 18′ 38.96″	75º 45′ 58.71″	1622	1901-1950
Kulgam	33º 38′ 40.59″	75º 01′ 04.91″	1746	1902-1950
Langet	34º 18′ 22.97″	74º 15′ 43.17″	1722	1916-1946
Poonch	33º 45′ 17.03″	74º 05′ 32.89″	981	1907-1950
Srinagar	34º 04′ 47.12″	74º 47′ 48.96″	1589	1893-2014

Supplementary Table 4. Calibration and verification statistics of different nests

S. No.	Proxy nests	Number of series	Calibration		Verification				
			Period	ar^2 [%]	Period	R	Sign test	RE	CE
1	1717-2014	5	1907-1926	50	1927-1946	0.69 (0.00076)	$17^{+}/3^{-}$ (0.0026)	0.40	0.53
			1927-1946	44	1907-1926	0.73 (0.00026)	$18^{+}/2^{-}$ (0.0004)	0.45	0.47
			1907-1946	49					
			1907-1946 L1	44					
2	1595-2005	4	1907-1926	50	1927-1946	0.68 (0.00097)	$15^{+}/5^{-}$ (0.04)	0.43	0.52
			1927-1946	43	1907-1926	0.72 (0.00034)	$18^{+}/2^{-}$ (0.0004)	0.48	0.46
			1907-1946	48					
			1907-1946 L1	43					
3	1595-2010	3	1907-1926	47	1927-1946	0.66 (0.0015)	$14^{+}/6^{-}$ (0.11)	0.42	0.50
			1927-1946	40	1907-1926	0.70 (0.00059)	$17^{+}/3^{-}$ (0.0026)	0.45	0.43
			1907-1946	45					
			1907-1946 L1	40					
4	1595-2011	2	1907-1926	44	1927-1946	0.65 (0.0019)	$15^{+}/5^{-}$ (0.04)	0.40	0.50
			1927-1946	39	1907-1926	0.70 (0.00059)	$17^{+}/3^{-}$ (0.0026)	0.45	0.42
			1907-1946	44					
			1907-1946 L1	40					

ar^2- r^2 adjusted for reduction ofdegrees of freedom, R- Pearson correlation coefficient, Sign test, RE (reduction of error) and CE (coefficient of efficiency), L1- cross-validation using Leave-one-out method[1,2]. The two tailed p values are given in brackets.

Supplementary Table 5. Wet/dry periods based on running means of 11, 21, and 31 year z-score values of SPI2-May.

11-year mean				21-year mean				31-year mean			
wet		Dry		wet		dry		wet		dry	
years	z-score	years	z-score	years	z-score	years	z-score	years	z-score	years	z-score
1896-06	1.29	1480-90	-1.88	1994-14	1.15	1477-97	-1.82	1984-14	1.03	1466-96	-1.71
1948-58	1.28	1449-59	-1.73	1891-11	1.02	1448-68	-1.61	1811-41	0.96	1499-29	-0.95
2004-14	1.27	1491-01	-1.55	1947-67	0.96	1498-18	-1.04	1894-24	0.94	1532-62	-0.91
1989-99	1.17	1465-75	-1.54	1808-28	0.95	1536-56	-0.97	1939-69	0.77	1609-39	-0.70
1811-21	1.15	1504-14	-1.09	1835-55	0.87	1618-38	-0.94				
1832-42	1.02	1623-33	-1.05								
1921-31	1.01	1553-63	-1.05								

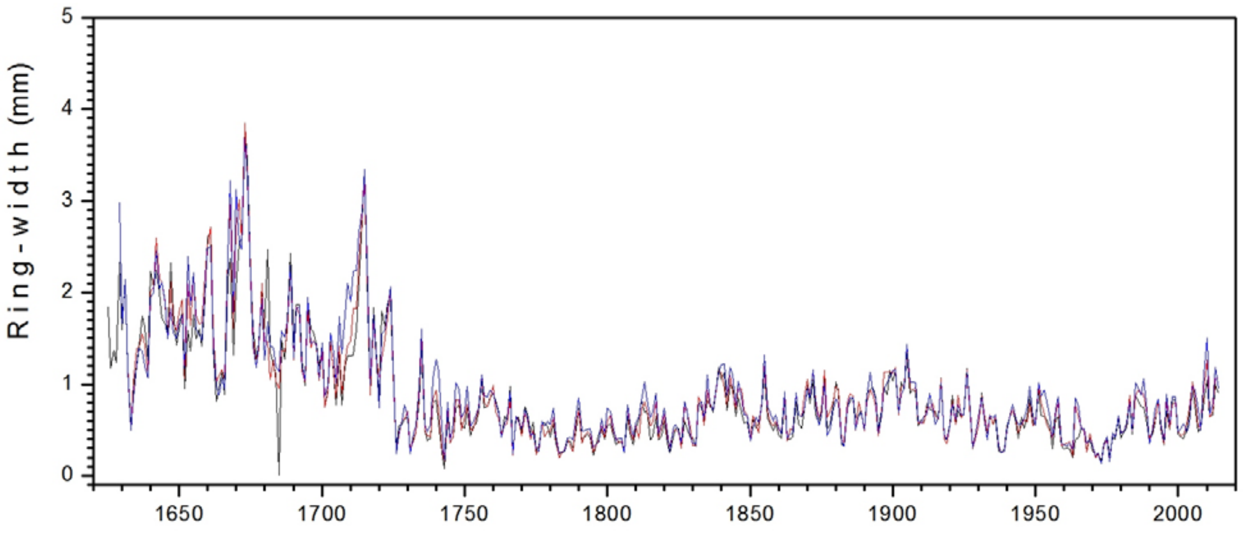

Supplementary Figure 1. Ring width measurement plot of a Neoza pine tree growing isolated on a steep rocky slope with extremely thin soil cover. Such trees growing in open conditions have a maximum common climate signal as disturbances due to competition and light are minimum. We preferred to collect samples from such natural, undisturbed trees. The tree shows a distinct increase in growth since the 1970s.

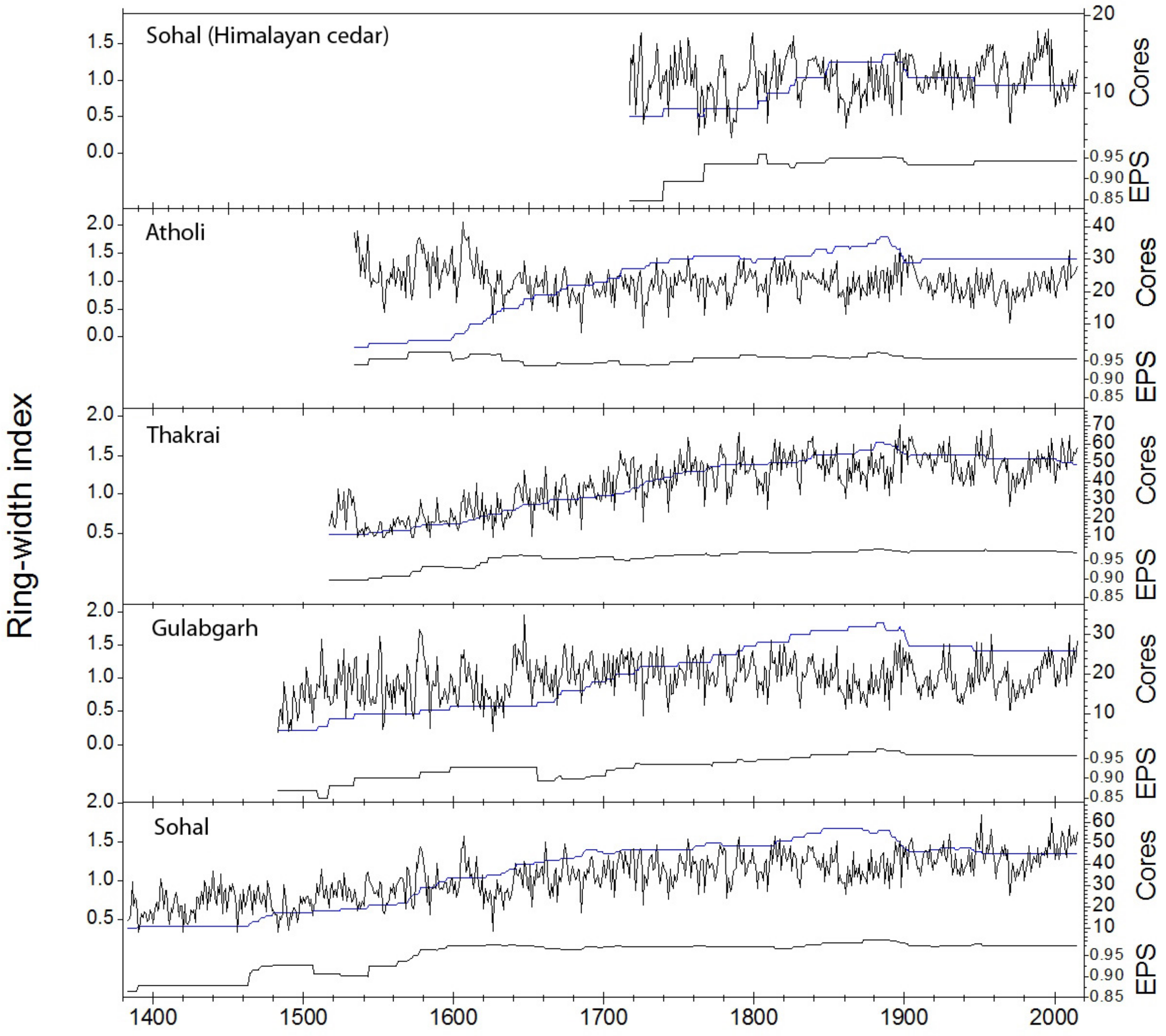

Supplementary Figure 2. Ring width chronologies of Neoza pine and Himalayan cedar from different sites prepared using the spline detrending method. The chronology panels are arranged from top to bottom with increasing length of chronologies. The chronologies are of Neoza pine except that of Himalayan cedar from Sohal shown in top panel. The expressed population signal statistics and number of cores used in chronology preparation are also indicated.

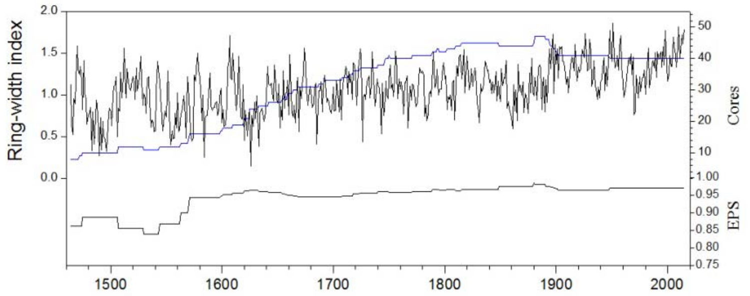

Supplementary Figure 3. Ring-width chronology of Neoza pine prepared using the Regional Curve standardization method. The number of cores used to calculate the chronology and EPS statistics are also indicated.

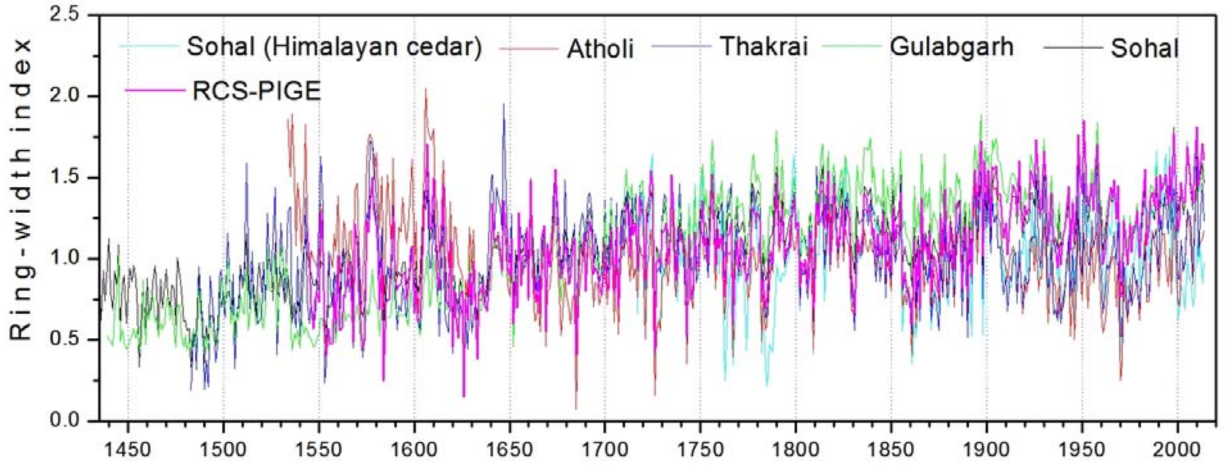

Supplementary Figure 4. Site and species chronologies plotted together to show year-to-year coherence in chronologies endorsing common external forcing affecting tree growth. Neoza pine (*Pinus gerardiana*) is abbreviated as PIGE.

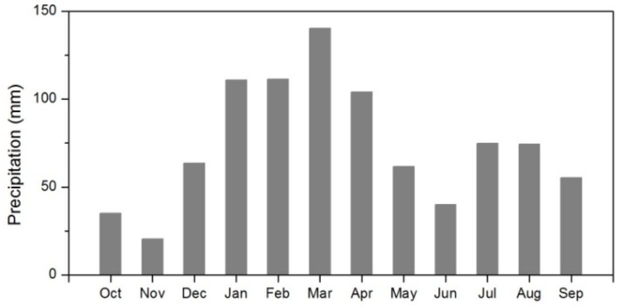

Supplementary Figure 5. Mean monthly precipitation (mm) of Kishtwar, Jammu and Kashmir (1901-1950)

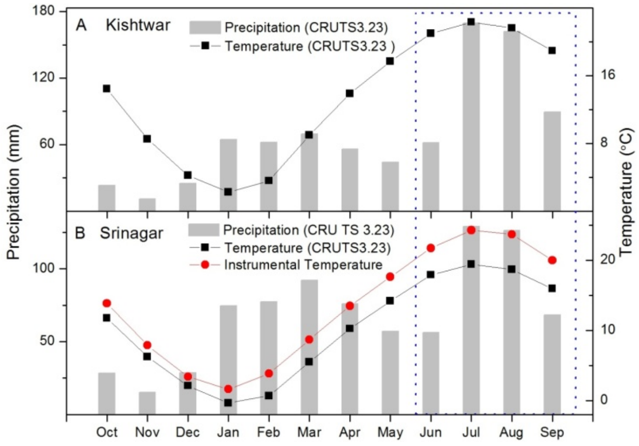

Supplementary Figure 6. Monthly distribution of temperature and precipitation data of grids (CRU TS3.23) close to Kishtwar (A) and Srinagar (B). Note that in situ weather data of Kishtwar, as shown in Supplementary Figure 5, show very different patterns in monthly precipitation patterns with that observed in gridded precipitation data of Kishtwar. However, the gridded data of Kishtwar and Srinagar show very similar pattern. Temperature data of Srinagar, both observed and gridded, show very similar patterns.

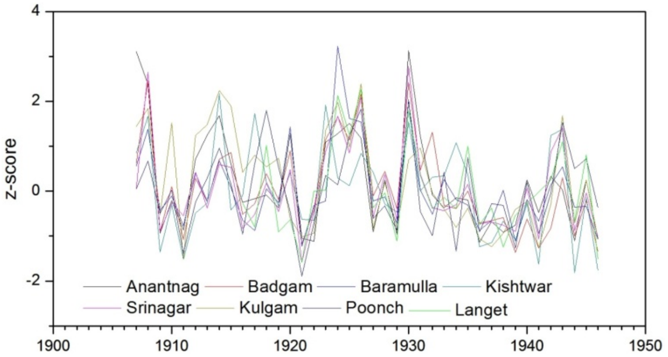

Supplementary Figure 7. April-May precipitation patterns of eight meteorological stations used to prepare mean regional precipitation series. Close year-to-year similarity in temporal variations in precipitation is notable.

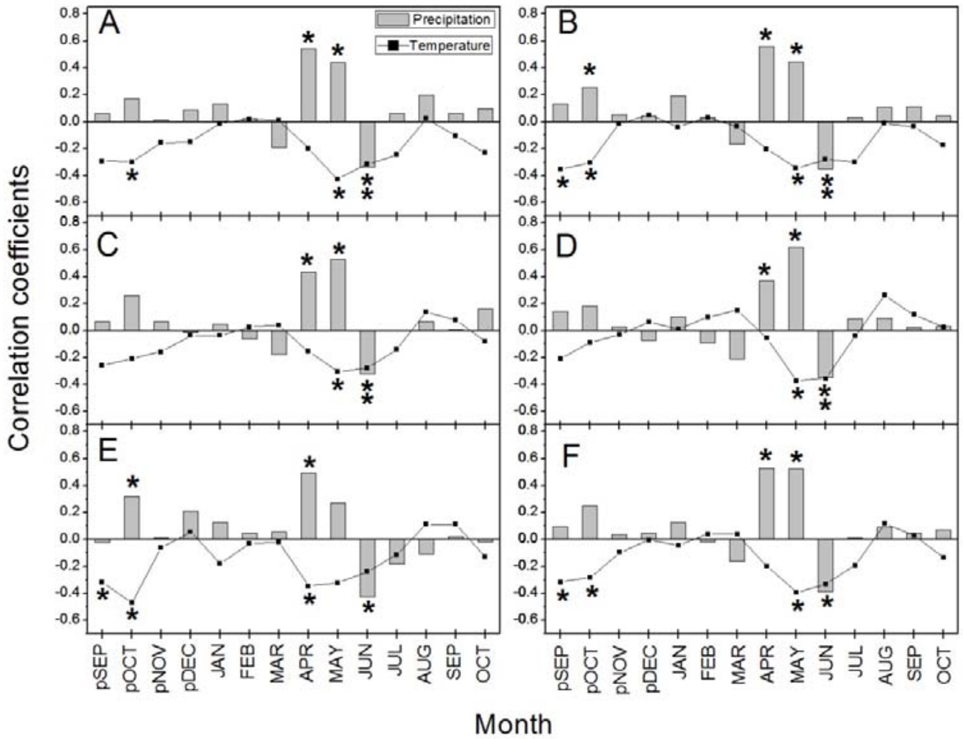

Supplementary Figure 8. Bootstrap correlation analyses between ring width chronologies of Neoza pine (A-Sohal, B-Gulabgarh, C-Thakrai, D-Atholi), Himalayan cedar (E-Sohal), PC#1 of all the chronologies (F) and monthly climate variables; regional mean precipitation series and temperature of Srinagar were used in the analyses. The bootstrap correlations were calculated using DENDROCLIM2002[3]. Correlations significant at $p<0.05$ are marked by an asterisk. Correlations were calculated for 1907-1946 when precipitation data of seven stations were available.

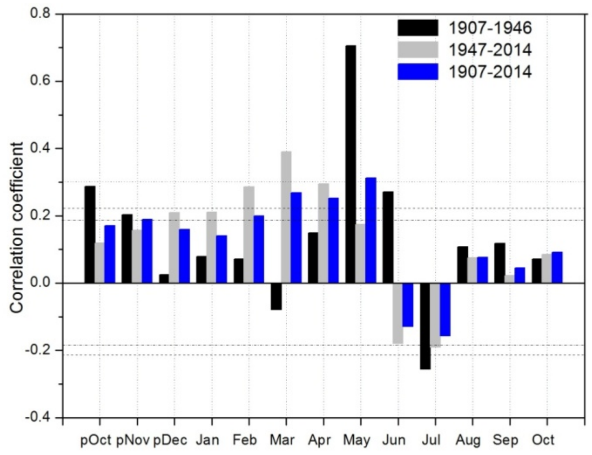

Supplementary Figure 9. Correlation between PC#1 and SPI2-May in different sub-periods; 1907-1946 when climate data of seven weather stations were available, 1947-2014 when only Srinagar precipitation data were used in SPI calculation, and for the whole period 1907-2014. Correlations significant at $p<0.05$ are indicated by dotted, dashed and dash-dotted lines for 1907-1946, 1947-2014 and 1907-2014 respectively.

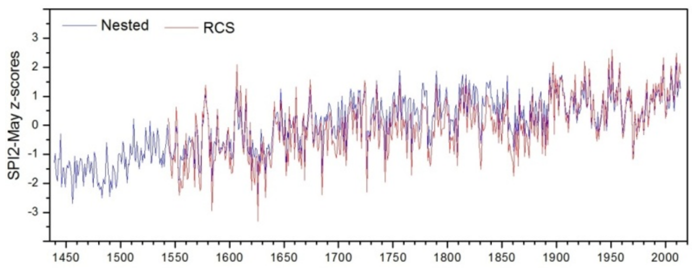

Supplementary Figure 10. SPI2-May reconstruction developed using a nested approach and the RCS chronology. Close similarity in increasing trend in reconstructed SPI developed using two independent methods is notable.

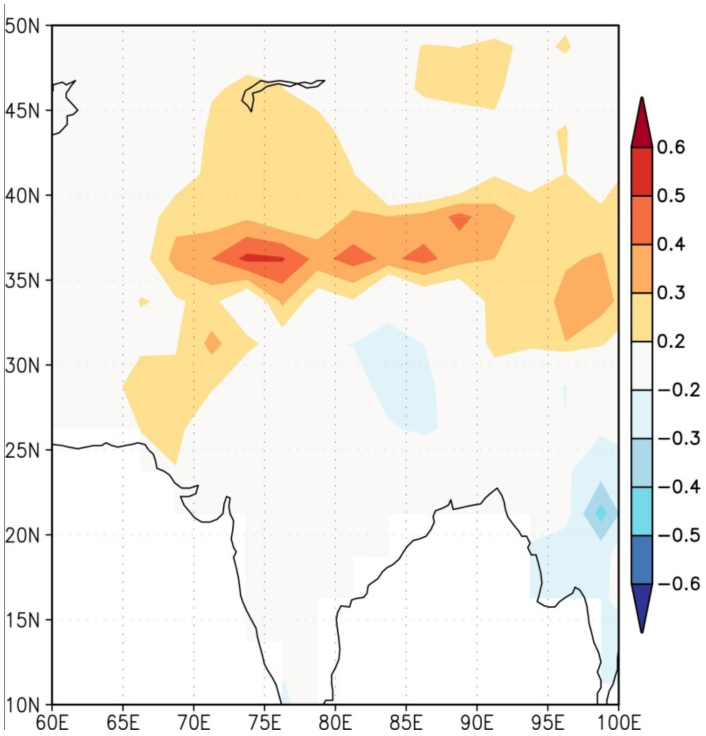

Supplementary Figure 11. Spatial correlation between reconstructed SPI2-May and scPDSI[4] (http://www.cgd.ucar.edu/cas/catalog/climind/pdsi.html) of April-May (1900-2014). The figure was generated using Climate Explorer Program (https://climexp.knmi.nl[5]).

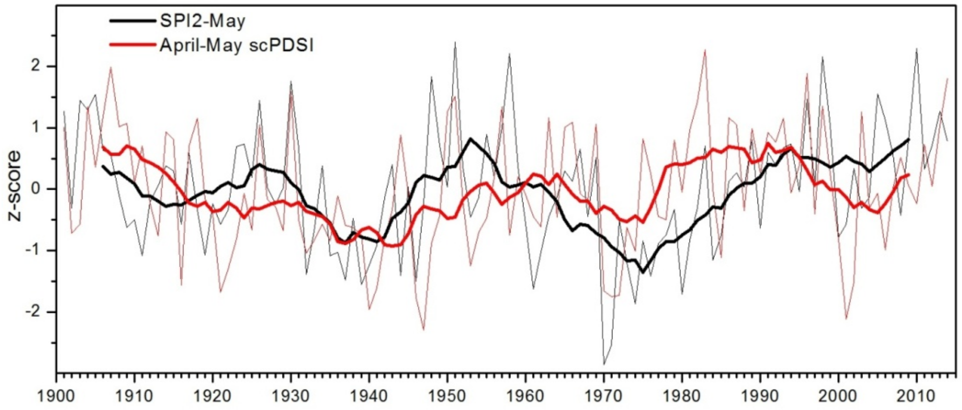

Supplementary Figure 12. SPI2-May and April-May scPDSI[4] (http://www.cgd.ucar.edu/cas/catalog/climind/pdsi.html) of the grid close to Kishtwar, Jammu and Kashmir (India) (33°-34°N and 74.5°-76°E) plotted together with an 11-year running average line to show coherence on inter annual-to-decadal time scales. The two series also showed significant Pearson correlation (r= 0.43, p< 0.0001, 1901-2014).

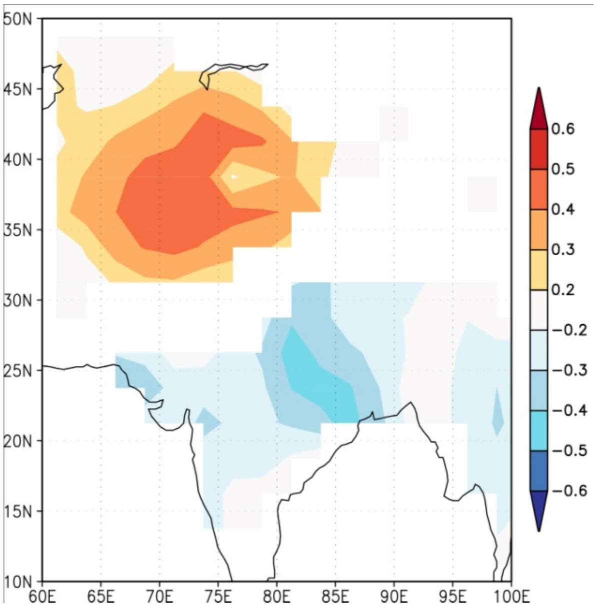

Supplementary Figure 13. Spatial correlation between reconstructed SPI2-May and gridded June-July-August PDSI of Monsoon Asia Drought Atlas[6] (http://www.ncdc.noaa.gov/paleo/pubs/cook2010/cook2010.html) for A. D. 1700-2005. Correlations back to A. D. 1700 were also similar as noted for the above period but weakened gradually back in time. The figure was generated using Climate Explorer Program (https://climexp.knmi.nl[5]).

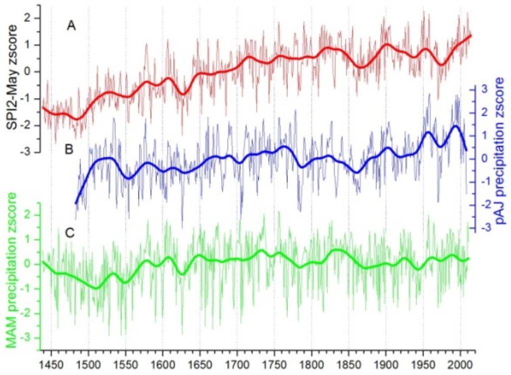

Supplementary Figure 14. Hydroclimatic reconstructions developed from the northwest Himalaya overlaid with respective 40-year low pass filters (thick lines) plotted to show relationship. A- SPI2-May reconstruction (A. D. 1439-2014), B- previous year August-current year July (pAcJ) precipitation reconstruction for Lahaul, NW Himalaya[7], C-March-April-May precipitation reconstruction for Kinnaur, NW Himalaya[8].

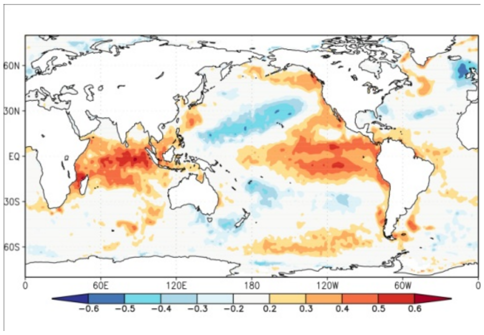

Supplementary Figure 15. Spatial correlation analyses between reconstructed SPI2-May and gridded Hadley Centre SST data set (HadlSST1)[9] (http://hadobs.metoffice.gov.uk/hadisst/) of corresponding months April-May for A. D. 1970-2014. The figure was generated using Climate Explorer Program (https://climexp.knmi.nl[5]).

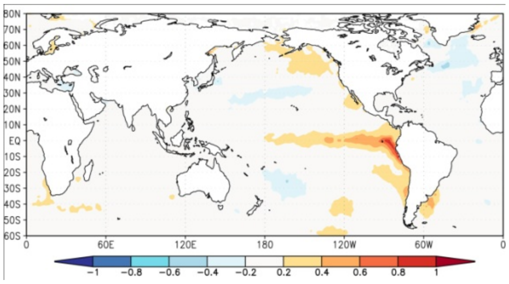

Supplementary Figure 16. Composite map for SPI2-May with gridded April-May gridded SST fields (1901-2014). The Hadley Centre SST data set (HadlSST1)[9] (http://hadobs.metoffice.gov.uk/hadisst/) were used in the analyses. Composite map was generated for SPI2-May >1 over the period 1901-2014. Colored shadings represent averaged detrended SST anomaly. Climate Explorer Program (https://climexp.knmi.nl[5]) was used to generate the picture.

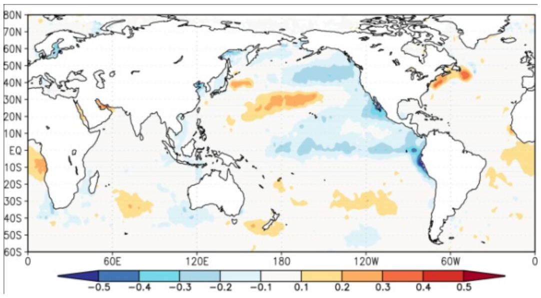

Supplementary Figure 17. Composite map for SPI2-May with gridded April-May gridded SST fields (1901-2014). The Hadley Centre SST data set (HadlSST1)[9] (http://hadobs.metoffice.gov.uk/hadisst/) were used in the analyses. Composite map was generated for SPI2-May < -1 over the period 1901-2014. Colored shadings represent averaged detrended SST anomaly. The figure was generated using Climate Explorer Program (https://climexp.knmi.nl[5]).

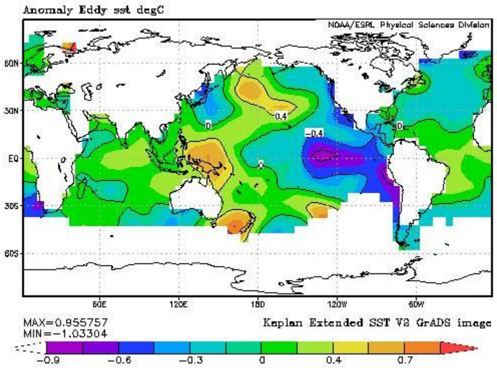

Supplementary Figure 18. Kaplan global SST anomaly plot for April 1970 to May 1971. The SST anomalies are relative to 1951-1980 mean. Kaplan SST V2 data[10] provided by the NOAA/OAR/ESRL PSD, Boulder, Colorado, USA were used to generate this figure using the link http://www.esrl.noaa.gov/psd/data/gridded/data.kaplan_sst.html.

References

1. Fritts, H.C. Tree-rings and Climate. Academic Press, London (1976).

2. Cook, E. R., Meko, D. M., Stahle, D. W.& Cleaveland, M. K., 1999. Drought reconstruction for the continental United States. *J. Clim.* **12**, 1145-1162 (1999).

3. Biondi, F. & Waikul, K. DENDROCLIM2002: A C++ program for statistical calibration of climate signals in tree-ring chronologies. *Comput. Geosc.* **30**, 303-311 (2004).
4. van der Schrier, G., Barichivich, J., Briffa, K. R. & Jones, P. D. A scPDSI-based global data set of dry and wet spells for 1901–2009. *J. Geophys. Res., Atmospheres* **118**, 4025-4048 (2013).
5. Oldenborgh, G. J. & Burgers, G. Searching for decadal variations in ENSO precipitation teleconnections. *Geophys. Res. Lett.* **32**, L15701, doi: 10.1029/2005GL023110 (2005).
6. Cook, E. R. *et al.* Asian monsoon failure and megadrought during the last millennium. *Science* **328**, 486-489 (2010).
7. Yadav, R. R. Tree-ring evidence of 20th century precipitation surge in monsoon shadow zone of western Himalaya, India, *J. Geophys. Res., Atmospheres* **116**, D02112, doi:10.1029/2010JD014647 (2011).
8. Yadava, A. K., Braeuning, A., Singh, J. & Yadav, R. R. Boreal spring precipitation variability in the cold arid western Himalaya during the last millennium, regional linkages, and socio-economic implications. *Quat. Sci. Rev.* **144**, 28-43 (2016).
9. Rayner, N. A. *et al.* Global analyses of sea surface temperature, sea ice, and night marine air temperature since the late nineteenth century. *J. Geophys. Res., Atmospheres* **108,** D14, 4407, doi: 10.1029/2002JD002670 (2003).
10. Kaplan, A., Cane, M., Kushnir, Y., Clement, A., Blumenthal, M., & Rajagopalan, B. Analyses of global sea surface temperature 1856-1991 *J. Geophys. Res., Oceans* **103**, 18567-18589 (1998).
